# Effect of scanning speed on texture-elicited vibrations

**DOI:** 10.1098/rsif.2019.0892

**Published:** 2020-06-10

**Authors:** Charles M. Greenspon, Kristine R. McLellan, Justin D. Lieber, Sliman J. Bensmaia

**Affiliations:** 1 Department of Organismal Biology and Anatomy, University of Chicago, Chicago, IL, USA; 2 Committee on Computational Neuroscience, University of Chicago, Chicago, IL, USA; 3 Grossman Institute of Neuroscience, Quantitative Biology, and Human Behavior, University of Chicago, Chicago, IL, USA

**Keywords:** touch, neural coding, haptics, somatosensory coding, vibrotaction

## Abstract

To sense the texture of a surface, we run our fingers across it, which leads to the elicitation of skin vibrations that depend both on the surface and on exploratory parameters, particularly scanning speed. The transduction and processing of these vibrations mediate the ability to discern fine surface features. The objective of the present study is to characterize the effect of changes in scanning speed on texture-elicited vibrations to better understand how the exploratory movements shape the neuronal representation of texture. To this end, we scanned a variety of textures across the fingertip of human participants at a variety of speeds (10–160 mm s^−1^) while measuring the resulting vibrations using a laser Doppler vibrometer. First, we found that the intensity of the vibrations—as indexed by root-mean-square velocity—increases with speed but that the skin displacement remains constant. Second, we found that the frequency composition of the vibrations shifts systematically to higher frequencies with increases in scanning speed. Finally, we show that the speed-dependent shift in frequency composition accounts for the speed-dependent change in intensity.

## 1. Introduction

To discern the texture of a surface, we spontaneously run our fingers across it [1], an exploratory procedure that results in the elicitation of skin vibrations that reflect the microstructure of the surface. While coarse textural features can be sensed without movement, our perception of fine textural features relies on the processing of skin vibrations elicited during scanning [2–5]. Two populations of tactile nerve fibres—rapidly adapting (RA) and Pacinian (PC) fibres—are exquisitely sensitive to skin vibrations and produce millisecond-precision temporal spiking sequences that reflect the vibrations [3,4,6,7]. These vibration-sensitive afferents mediate our ability to perceive fine textural features as evidenced by the fact that desensitizing them impairs the perception of fine texture [5].

Texture-elicited vibrations are sensitive not only to surface microstructure but also to exploratory movements, particularly scanning speed. Indeed, skin vibrations dilate or contract with decreases or increases in scanning speed [7,8], respectively, resulting in concomitant dilations or contractions of the evoked spiking sequences in the nerve [6]. In addition, the firing rates of nerve fibres and of their downstream targets tend to increase with increases in scanning speed [9,10]. Whether this enhanced neural response reflects the contraction of the spike trains or is caused by an additional increase in the amplitude of the vibrations—which would also lead to higher firing rates—remains to be elucidated.

In a previous study, the effect of scanning speed on texture-elicited vibrations was examined by scanning surfaces across the skin at a single speed within each experimental block and measuring the evoked vibrations using a laser Doppler vibrometer (LDV) [7]. Different speeds were tested on different blocks. When measuring skin vibrations using an LDV, the overall gain depends on the angle of incidence of the laser beam and the distance between its focus and the point of contact with the surface [11]. Thus, the gain of the measurements varied from block to block, so the raw magnitude of the vibrations could not be compared across experimental blocks; therefore, the effect of speed on vibratory intensity could not be assessed. To fill this gap, we used an LDV to measure the vibrations evoked in the skin when everyday textures are scanned across the skin over a range of behaviourally relevant speeds within single blocks [12].

We found (as expected) that the frequency composition of vibrations shifts to higher or lower frequencies with increases or decreases in scanning speed, respectively. Furthermore, vibratory intensity, as indexed by root-mean-square (RMS) velocity, increases with speed but does so in a texture-specific way. We then demonstrated that these texture-specific changes in vibratory intensity can be explained by the multiplicative shift in the frequency composition of the vibrations. We discuss the implications of these findings for the neural coding of both texture and speed.

## 2. Results

Using a custom-built rotating drum stimulator (see [7]), we scanned eight textured surfaces across the right index fingertip of five human subjects (two males and three females, age range 20–25 years) at 28 speeds spanning the range used in natural texture exploration (ranging from 10 to 160 mm s^−1^) [12] while measuring the evoked skin vibrations using an LDV (OFV-3001 with OFV 311 sensor head; Polytec, Irvine, CA).

### 2.1. Effect of scanning speed on the intensity of texture-elicited vibrations

**Figure 1.**
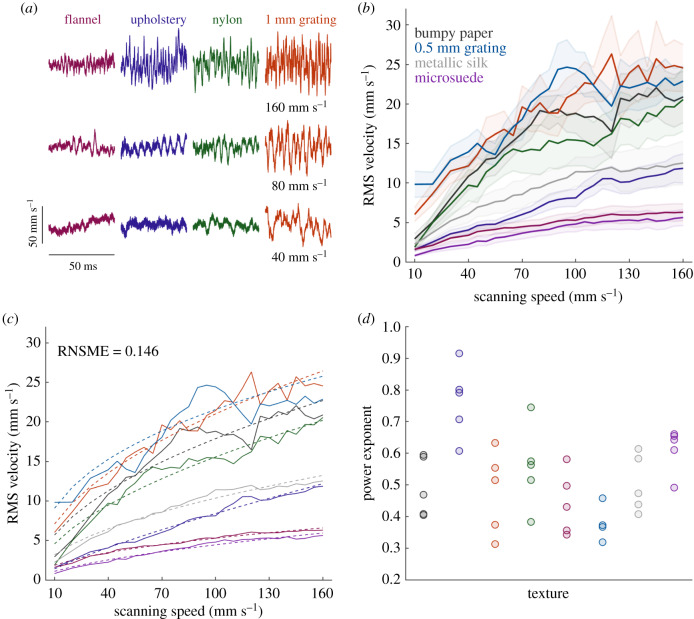
The RMS velocity of texture-elicited vibrations increases as scanning speed increases. (*a*) Example velocity traces for four textures scanned at three speeds. (*b*) RMS velocity elicited by each texture versus speed; solid line and shaded area indicate mean and standard error, respectively. (*c*) Mean measured RMS velocity (solid line) and the power model prediction (dashed line). Each trace denotes a different texture. (*d*) Fitted exponents for each texture and participant.

First, we found that the intensity of the vibrations elicited in the skin, as indexed by RMS velocity, varied widely across textures (figure 1*a,b*) (three-way ANOVA, *F*_7,1118_ = 1280.08, *p* < 0.001, *η*^2^ = 0.51969), as has been previously shown in [7]. Indeed, RMS velocity varied over more than an order of magnitude at both the slowest scanning speed (0.511–13.3 mm s^−1^ at 10 mm s^−1^) and the fastest one (2.08 and 33.2 mm s^−1^ at 160 mm s^−1^). Second, we found that RMS velocity increased systematically with scanning speed (*F*_27,1118_ = 106.01, *p* < 0.001, *η*^2^ = 0.16960), but in a texture-dependent way, as evidenced by a significant texture × speed interaction (*F*_189,1118_ = 3.92, *p* < 0.001, *η*^2^ = 0.0430). Indeed, RMS velocity increased 2- to 11-fold from the lowest to the highest scanning speed, depending on the texture. The relationship between velocity and scanning speed was well described by a power law, with different parameters—exponent and scaling factor—for different textures (figure 1*c*,*d*). Mean exponents ranged from 0.43 to 0.58 across textures. RMS velocity did not just vary across textures but also across participants (*F*_4,1118_ = 449.24, *p* < 0.001, *η*^2^ = 0.1042). The participant × speed interaction was weak but statistically significant (*F*_108,1118_ = 2.34, *p* < 0.001, *η*^2^ = 0.0147).

**Figure 2.**
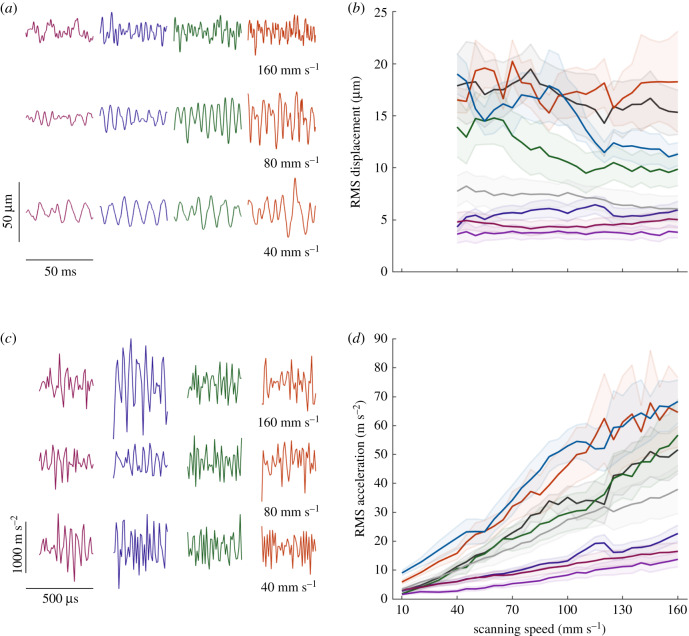
Effect of scanning speed on RMS displacement and acceleration. (*a*) Example displacement traces for four textures scanned at three speeds. (*b*) RMS displacement for each texture between 40 and 160 mm s^−1^. Speeds below 40 mm s^−1^ are discarded owing to noise (see Methods). (*c*) Example acceleration traces for four textures scanned at three speeds. (*d*) RMS acceleration for each texture across all speeds. Lines and shaded areas for (*b*,*c*) represent mean and standard error, respectively. Color scheme follows that of figure 1.

Next, we examined the effect of scanning speed on the displacement and acceleration profiles of the texture-evoked vibrations (the integral and derivative of velocity, respectively). RMS displacement was primarily dependent on texture (*F*_7,999_ = 1077.26, *p* < 0.001, *η*^2^ = 0.6542), reflecting differences in the size of the textural features across surfaces (figure 2*a*,*b*). By contrast to RMS velocity, however, displacement either remained constant or decreased slightly with increases in scanning speed, depending on the texture, and the overall effect of speed on RMS displacement was small (*F*_24,999_ = 5.25, *p* < 0.001, *η*^2^ = 0.0109) (electronic supplementary material, figure 1*a*,*b*). RMS acceleration (figure 2*c*,*d*) increased nearly linearly with speed at a rate that was texture dependent (electronic supplementary material, figure 1*c*,*d*). While texture was the dominant source of variance in RMS acceleration (*F*_7,1118_ = 825.25, *p* < 0.001, *η*^2^ = 0.3629), speed was also highly significant (*F*_27,1118_ = 163.21, *p* < 0.001, *η*^2^ = 0.2768).

If the skin vibrations elicited during scanning simply reflected the surface profile, then RMS displacement would be constant across speeds, RMS velocity would increase linearly with speed and RMS acceleration would increase supra-linearly with speed (electronic supplementary material, figure 2). Our results do not exactly match this prediction because the assumption that displacement is constant is violated for some textures (see below).

### 2.2. Effect of scanning speed on the frequency composition of texture-elicited vibrations

**Figure 3.**
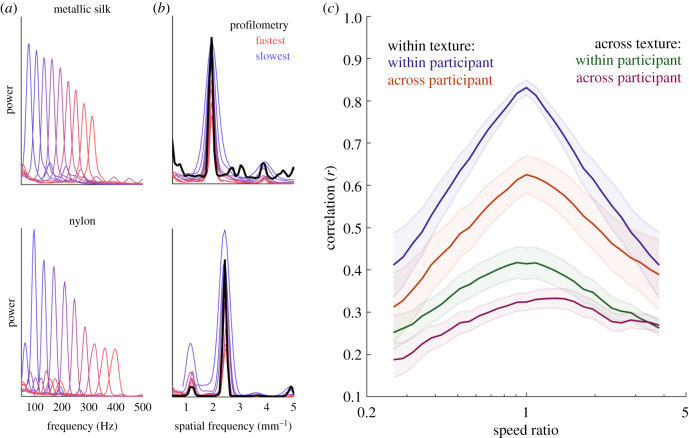
Spectral profile of the vibrations is shifted in the temporal domain but conserved in the spatial domain. (*a*,*b*) The frequency composition of the vibrations (expressed as displacements) shifts to higher frequencies with increases in scanning speed but remains relatively consistent when expressed in spatial units. (*c*) Correlations within or across participants and/or textures versus speed ratio. The correlation decreases as the difference in speed increases. Color scheme follows that of figure 1.

Next, we examined how the frequency composition of the vibrations changed with scanning speed. We observed that, consistent with previous findings, the power spectral density (PSD) shifted systematically towards higher frequencies as scanning speed increased (figure 3*a*). The spectral profile of these vibrations, however, was relatively consistent across speeds when expressed in spatial units (by dividing the temporal frequency by the speed; figure 3*b*) (cf. [7,8,13]). To quantify the consistency of the spectral profile across speed, we computed the correlation of the PSD across pairs of speeds for each texture and participant. First, we found that the correlation across repeated presentations of the same texture at the same speed was very high (mean ± s.e.m: *r* = 0.832 ± 0.017), highlighting the repeatability of the measurements themselves. Second, PSDs obtained from a given texture and participant remained similar even across large changes in speed (figure 3*c*). Indeed, when speed was doubled or halved, the mean correlation dropped from 0.82 to 0.65. Third, across-subject/within-texture PSD were similar, though less so than their within-subject counterparts, and correlations exhibited a similar drop-off as speed difference increased. Non-zero correlations when comparing across-texture PSDs revealed coincidental similarities in the vibrations elicited by certain pairs of surfaces, driven mostly by aperiodic textures that feature a prominent 1/*f* drop-off in their frequency composition.

**Figure 4.**
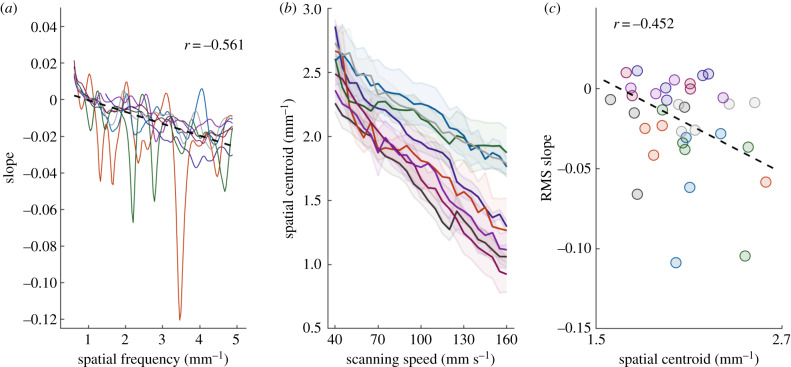
The amplitude of high-frequency components tends to decrease when scanning speed increases. (*a*) The ratio between the expected and observed peak power for a PSD is scanning speed dependent. High spatial frequency components decrease in power at a greater rate than do low spatial frequency ones. (*b*) The centroid of the PSD in spatial units shifts systematically downwards as the speed is increased. (*c*) The frequency composition of the PSD partly predicts how the RMS displacement will change with speed (spatial centroids are for 90 mm s^−1^). Color scheme follows that of figure 1; dashed black line in (*a*,*c*) represents the line of best fit.

Having established that the (spatial) PSDs were similar but not identical across speeds, we then examined *how* the shape of the PSD changed with speed. Examination of the spatial PSDs revealed that textural components at higher spatial frequencies tended to be preferentially suppressed as speed increased; that is, the skin was less prone to faithfully follow surface components at high spatial frequencies when the surface moved rapidly across the skin (figure 3*b*). To quantify this phenomenon, we split the displacement PSD, expressed in terms of spatial frequency, into frequency bands for each texture, and computed the change in power within each frequency band (figure 4*a*). As expected, high spatial frequency components tended to decrease with increases in speed, resulting in a decrease in the spatial frequency centroid for all textures (figure 4*b*). Furthermore, textures with high spatial frequency centroids tended to exhibit the greater decrement in RMS displacement with increases in scanning speed (figure 4*c*). In other words, the skin tends to follow the profile of the surface but does so better at low speeds and for textures with low spatial frequencies. This phenomenon can account for the discrepancy between the expected (electronic supplementary material, figure 2) and observed (figures 1 and 2) effects of speed on the texture-elicited vibrations.

## 3. Discussion

### 3.1. Variability across participants

While texture-elicited vibrations were similar across participants, they were not identical. Indeed, the speed dependence of the shape of the PSDs varied widely across participants and differences across participants accounted for over 10% of the variance in the RMS velocity. These differences are likely due to two factors. The first is that skin lubrication, which strongly modulates the frictional interactions between skin and texture, varies widely across individuals [14,15]. We attempted to minimize variations in lubrication by cleaning fingertips with an alcohol wipe, but this intervention is unlikely to have completely eliminated differences in lubrication. The second contributor to differences across subjects is variation in the biomechanics and microstructure of the skin. The mechanical properties of the skin—stiffness and elasticity—vary substantially across individuals and impact skin–surface interactions [16–18]. Similarly, fingerprint microgeometry famously differs across individuals and impacts the skin response to scanned textures [7,19–21]. These factors combine to give rise to subject-specific interactions with surfaces, which are then reflected in the skin vibrations.

### 3.2. Implications for tactile texture perception

Texture perception is highly independent of scanning speed and contact force despite the fact that the response of the skin and that of tactile nerve fibres is highly dependent on these exploratory parameters [6,22]. Indeed, the firing rates of tactile nerve fibres, particularly PC fibres, increase with increases in scanning speed. Our results suggest that the effect of speed on afferent responses is mediated by an increase in RMS velocity (cf. [3]), itself caused by a shift in the frequency composition of the vibrations to higher frequencies. Both the increase in RMS velocity of texture-evoked vibrations and the shift to higher frequencies explains why the effect of speed is most pronounced in the responses of PC fibres, weaker in RA fibres, and weakest in slowly adapting (SA1) fibres [23]. Indeed, PC fibres are sensitive to the rate of displacement of the skin [24] and peak in sensitivity at the high frequencies (greater than 100 Hz) [25]; RA fibres are also sensitive to vibratory speed but peak in sensitivity at intermediate frequencies; SA1 fibres are least sensitive to vibratory speed and peak in sensitivity at low frequencies. While the peripheral representation of texture is highly speed dependent, afferent signals are differentiated downstream—spatially and temporally—and these neural computations give rise to a more speed-independent representation of texture [26].

### 3.3. Implications for tactile speed perception

While we have a sense of how fast a surface is moving across our skin [23,27], tactile speed perception is powerfully biased by surface texture: coarser textures are systematically perceived as moving faster. Speed perception is not veridical and can be explained by its neural basis. Indeed, perceived speed is determined by the strength of the response evoked in PC fibres, itself dependent on both texture and scanning speed. In the present study, we replicate the finding that vibratory intensity—gauged with RMS velocity or acceleration—is strongly dependent on both surface texture and speed. Given that RMS acceleration at the high frequencies (greater than 50 Hz) is a good proxy for PC firing rates [28], the present results account for the dependence of perceived speed on both speed and texture [23].

### 3.4. Artificial textures

The rise of mobile devices—tablets and phones—has spurred the development of haptic interfaces to provide tactile feedback during manual interactions with the devices, most commonly in the form of vibrations triggered by contact with the screen. That texture perception relies in part on vibration has engendered attempts to elicit percepts by generating texture-like vibrations on the surface [29–34]. Because texture-elicited vibrations depend on both the texture and the exploratory parameters, a challenge in generating artificial textures has been to store the information necessary to replay vibrations appropriate for a given texture and set of exploratory parameters. One approach consists in tiling the space of possible scanning speeds and contact forces and interpolating between these measured values [33]. A parsimonious approach would be to store the vibratory profile of each texture at some intermediate speed (say 90 mm s^−1^) and warp said trace depending on the instantaneous scanning speed. The resulting vibrations would largely match their natural counterparts while relying on a single stored trace. One might improve upon this strategy by preferentially suppressing high spatial frequency components as scanning speed increases. Further improvements could be achieved by assessing the effects on the texture-elicited vibrations of contact force, which varies concomitantly with scanning speed [35] and modulates the responses of primary afferents to textures [22]. We expect this general approach to yield verisimilar textural percepts, at least to the extent that such percepts can be achieved given the current state of haptic technology.

## 4. Methods

### 4.1. Participants

Five subjects (two males and three females, 20–25 years of age) participated in the study. Procedures were approved by the Institutional Review Board of the University of Chicago.

### 4.2. Data collection

#### 4.2.1. Texture apparatus and stimuli

The subject sat with one arm resting on a padded frame. The hand was strapped to a custom hand holder with the palm facing upwards and the index finger propped at a 45° angle. The fingertip was cleaned with alcohol to minimize variations in skin moisture across participants. On each trial, a rotating drum stimulator (previously described in [7]) scanned one of eight or 11 texture strips (2.5 cm × 16 cm) across the subject's fingertip at a pre-calibrated contact force (30*g*) and at one of 28 speeds, ranging from 10 to 160 mm s^−1^, evenly spaced. Trial duration ranged from 0.4 s at the fastest speed to 1.6 s at the slowest one and the inter-trial duration was 3.5 s.

#### 4.2.2. Vibrometer

An LDV (Polytec OFV-3001 with OFV 311 sensor head; Polytec, Irvine, CA) was used to record the vibrations elicited in the skin (cf. [7,11]). Briefly, the vibrometer measures the time-varying velocity along the axis perpendicular to the surface of the skin, without making contact with it. A small strip of white-out correction fluid (BIC USA, Shelton, CT, USA) was applied to the distal aspect of the distal interphalangeal joint of the right index finger—near the location where the finger made contact with the surface—to improve the reflectance of the finger and reduce dropouts. The vibrometer beam was then directed and focused on the white-out.

### 4.3. Data analysis

#### 4.3.1. Preprocessing

The last 350 ms (35 000 data points) of each trace was used to exclude transients during the establishment of contact with the surface and ensure that the frequency resolution of the power spectra was equivalent across speeds. For each trace, outliers—which fell 6 SD from the mean for that speed/texture pair—were replaced by linear interpolation of the two adjacent time points. Dropout removal was performed iteratively until all were removed. Traces that contained 2 ms or more of dropouts were eliminated. Vibrations decay as they travel from their origin and do so in a frequency-dependent way (see [11]). We corrected for this decay based on previous measurements, assuming a distance of 1 cm between the measurement location and the nearest contact between skin and surface.

#### 4.3.2. Spectral analysis

Power spectral densities were computed using Welch's method over the range from 50 and 1000 Hz (see below). To convert frequency spectra to the spatial domain, we divided the frequency by the scanning speed (mm s^−1^). To compute displacement and acceleration, each component of the Fourier spectrum was divided or multiplied by 2*πf*, respectively, and the resulting spectra were converted back to the time domain. The RMS of the trace within the desired frequency band was then given by the following equation:

$$\text{RMS}=\text{sqrt}(\text{sum}(\text{PS}{\text{D}}_{\;f_{\mathrm{lfc}}-f_{\mathrm{hfc}}}\;*\;\text{Fr})),$$

where PSD is the power spectral density, *f* is the frequency, lfc*/*hfc are the frequency cut-offs and Fr is the frequency resolution of the PSD.

#### 4.3.3. Noise filtering

We wished to determine the range over which measured vibrations are texture specific. To this end, we first normalized each PSD so that it summed to 1 and calculated the mean normalized PSD across textures, speeds and participants (electronic supplementary material, figure 3*a*). We observed that the bulk of the vibratory energy was contained in the first 50 Hz. Next, we examined the degree to which the power in different frequency bands varied across textures (electronic supplementary material, figure 3*b*) and found that the low frequencies (up to approx. 50 Hz) were relatively texture independent, consistent with previous observations [7]. Finally, we performed the same analysis while varying both the low- and high-frequency cut-offs (electronic supplementary material, figure 3*c*) and found that performance was stable with high-frequency cut-offs above 300 Hz. Consequently, we used 50 Hz as the low-frequency cut-off and 1000 Hz as the high-frequency cut-off to ensure that no texture-dependent signals were discarded.

#### 4.3.4. Regression

We implemented three models to relate *RMS Velocity* to scanning speed: linear (RMS = *B* * speed), log-linear (RMS = *B* * log(speed)) and power (RMS = *B* * speed*^k^*), where *B* and *k* are free parameters. We did not include an intercept term as lack of movement across the skin should result in no vibration. We only report the results from the power relationship as it outperformed the other two. We found that the dependence of RMS Acceleration and RMS Displacement on speed was well captured by a linear relationship (RMS = *B* * speed + *o*). To assess the regression fits, we used the range-normalized mean squared error (RNMSE), given by the following equation:

$$\text{RNMSE}\;=\;\frac{\sqrt{\sum {\left(y\;-\;\hat{y}\right)}^{2}}}{y_{\max}\;-\;y_{\min}},$$

where *y* is the measured RMS and $\hat{y}$ is the predicted RMS.

#### 4.3.5. Spatial PSD analysis

To directly compare the PSDs at different speeds, we expressed them in spatial units by dividing frequency by speed, then resampling the PSDs via linear interpolation to achieve the same frequency resolution (matched to that of the reference speed). We then computed the correlations of pairs of PSDs (within texture/within participant, within texture/across participants, etc.). When determining the degree to which the power at each spatial frequency changed with speed, we used the same resampled PSDs and performed linear regression on the ratio of the power at each frequency relative to its power at the reference speed for each participant and texture pair and report the average value across participants. To calculate the spatial centroid, we computed the temporal centroid within the frequency band and then divided the resulting value by the speed.

## Supplementary Material

Supplementary Figures

## Supplementary Material

Vibrometry

## Data accessibility

Data available as part of the electronic supplementary material.
