# Supplementary material for: Effect of scanning speed on texture-elicited vibrations
Source: J R Soc Interface. 2020 Jun 10;17(167):20190892. doi: 10.1098/rsif.2019.0892 (PMC7328380; doi:10.1098/rsif.2019.0892)
Supplement: Supplementary Figures [file rsif20190892supp1.docx]

**Supplementary figures**


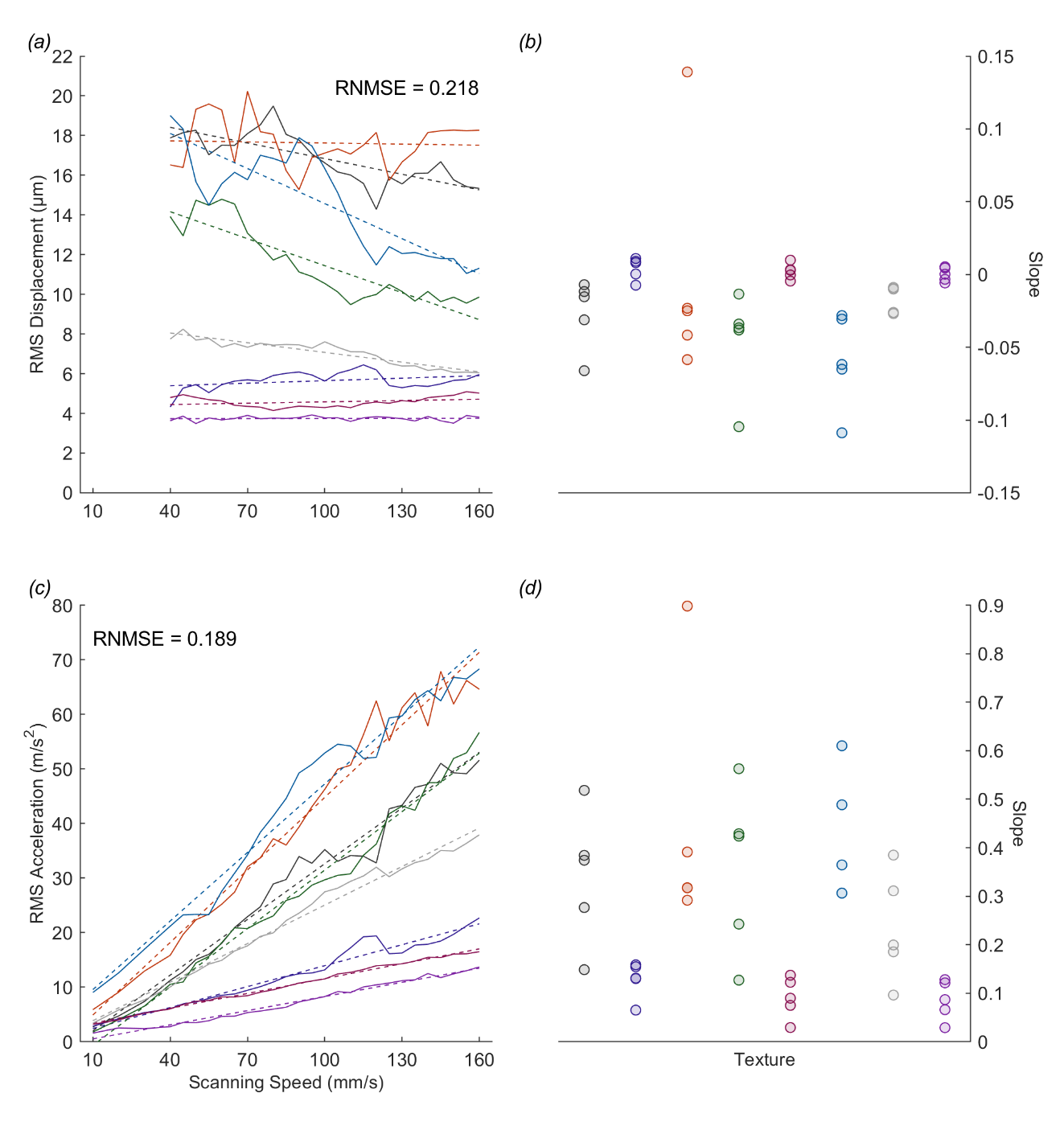


**Supplementary Figure 1 |** Effect of scanning speed on RMS displacement and acceleration. (a) Mean RMS displacement is constant or decreases slightly with speed. (b) Slope coefficient for each texture and participant. (c/d) RMS acceleration increases with scanning speed. Dashed lines show linear fits.


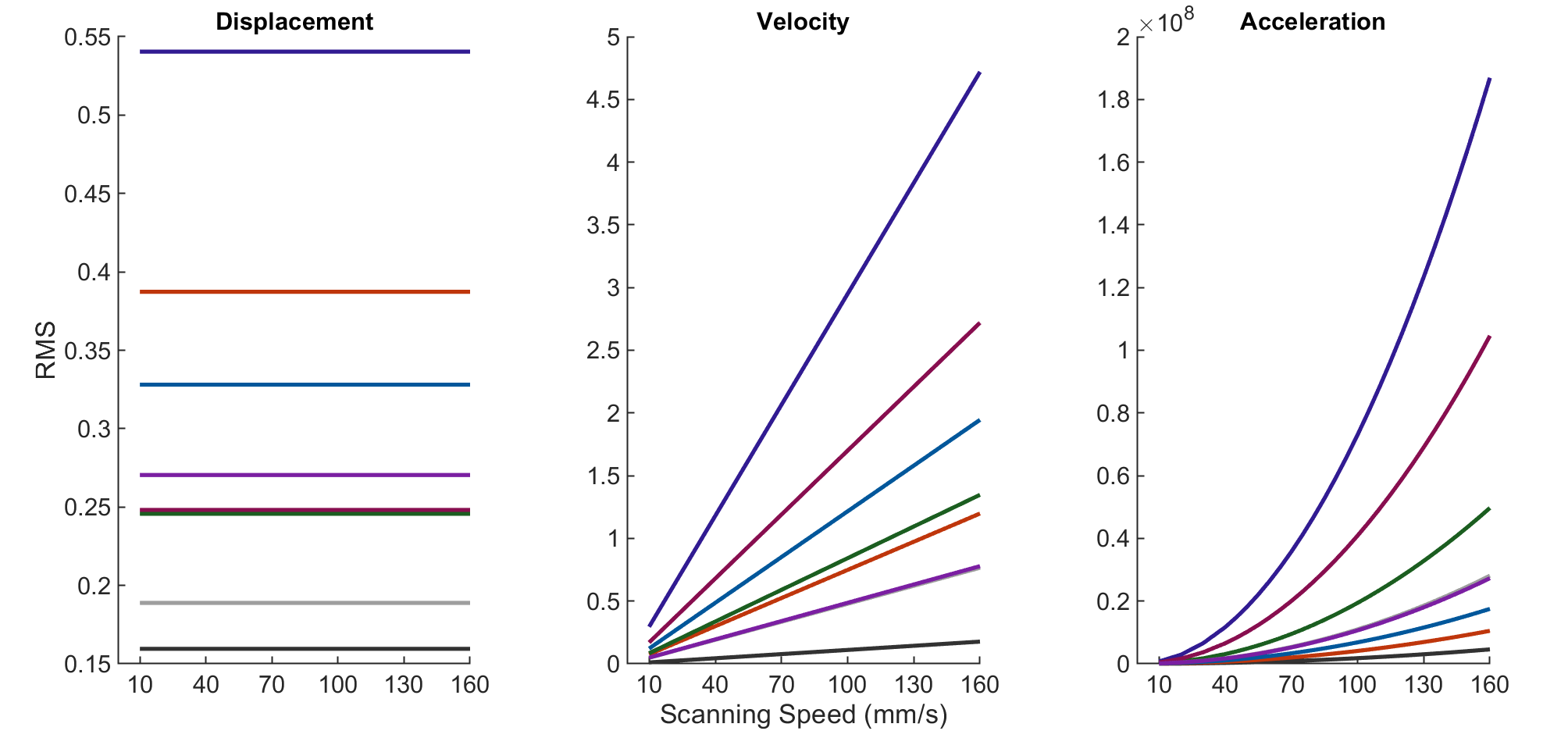


**Supplementary Figure 2 |** Predictions, computed from the profilometry, of RMS velocity and acceleration given constant displacement. If increases in scanning speed simply shift the power spectrum to higher frequencies, then RMS displacement will be speed-independent, and both RMS velocity and RMS acceleration will increase given that components are shifted to higher frequencies.

**
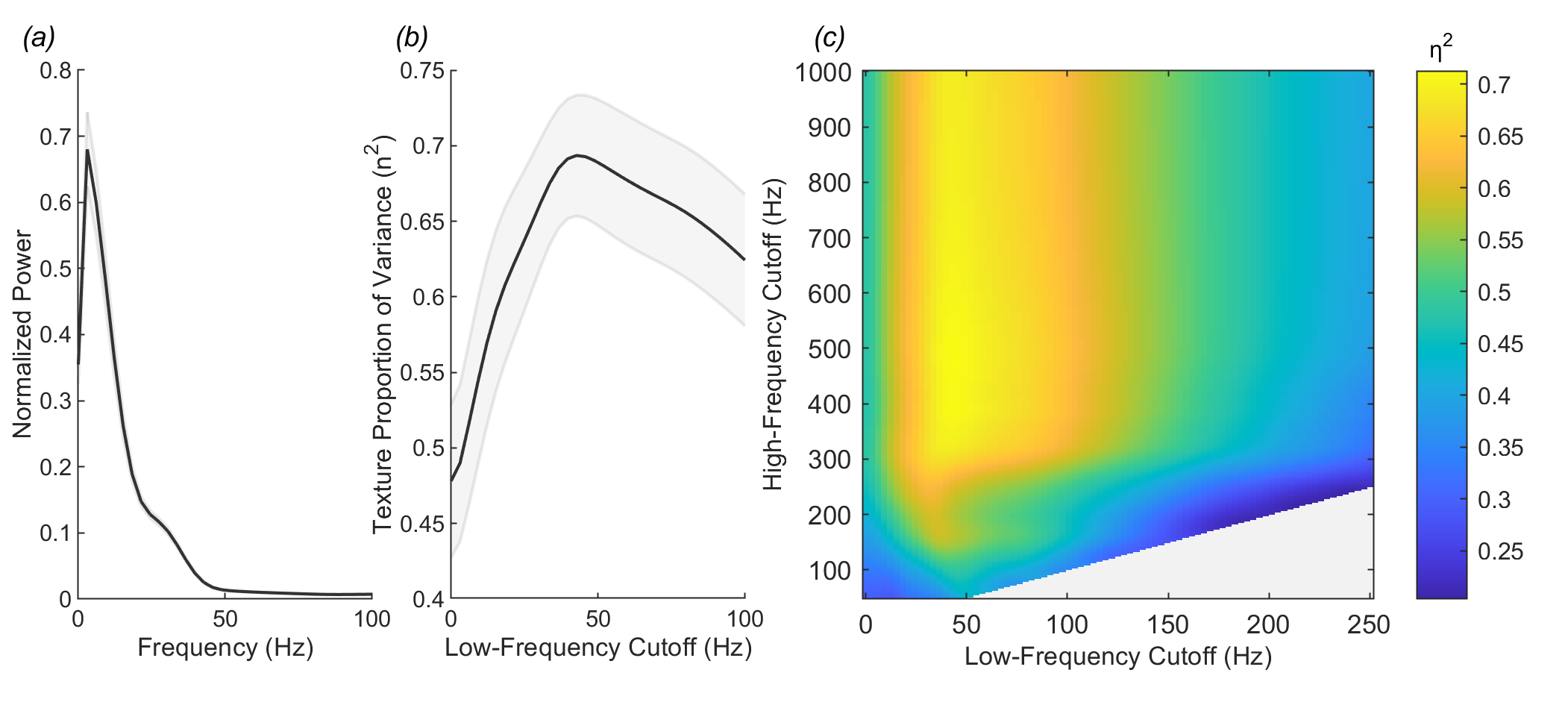
**

**Supplementary Figure 3 |** At low frequencies, noise dominates the signal and obscures texture-related information. (*a*) Average normalized power spectral density for all traces. Much of the power is concentrated at the low frequencies (*b*) Proportion of variance attributed to texture (computed using a 2-way ANOVA) increases as the low-frequency cutoff increases up to about 50 Hz. Black line is average and gray shaded area SEM. (*c*) The average proportion of variance explained by texture across participants when changing both the low- and high-frequency cutoff.
